# Data on birds and habitat associated with forest management on public conservation areas in the Mississippi Alluvial Valley

**DOI:** 10.1016/j.dib.2016.11.011

**Published:** 2016-11-09

**Authors:** Daniel J. Twedt, R. Randy Wilson

**Affiliations:** a U.S. Geological Survey, Patuxent Wildlife Research Center, University of Memphis, Memphis, TN 38152, USA; b U.S. Fish and Wildlife Service, 6578 Dogwood View Parkway, Suite B, Jackson, MS 39213, USA

**Keywords:** Birds, Hardwood forest, Wildlife forestry, Basal area, Public conservation land

## Abstract

This data article contains data collected from 2006–2012 in forests located on 31 State or Federal conservation lands in or adjacent to the Mississippi Alluvial Valley. We present the location, treatment type, and silvicultural age of data collection locations. Presented data on bird detections and forest habitat were collected during avian point counts and associated forest habitat plots and linked to the publication (D.J. Twedt and R.R. Wilson, 2017) [5].

**Specifications Table**

**Table:** 

Subject area	Biology
More specific subject area	Wildlife Management
Type of data	Table, figure, supplementary spreadsheet
How data was acquired	Avian point counts (time and distance categories within 10-minute intervals [2])
	Trees and habitat coverage within 10 basal area factor (BAF) prism plots [1]
Data format	Raw, summarized
Experimental factors	Forest stands were unharvested control stands or subjected to silvicultural harvests intended to enhance wildlife habitat [3]
Experimental features	Control stands had not been harvested for >20 years and included designated natural areas not subject to harvest. Treated stands were subjected to wildlife forestry silvicultural harvests from 1 to 20 years before data collection. Silvicultural harvests ranged markedly in extent and intensity
Data source location	Mississippi Alluvial Valley, southern USA
Data accessibility	Data are provided within this article

**Value of the data**

• Data provide location, relative intensity of silvicultural treatment, and age of treatment for use in evaluation of the distribution of wildlife forestry treatments.
• Categorical time, distance, and species of first detection of each individual bird provide information to evaluate detection probability [4] and detection distance sufficient to enable density estimates that can be compared with avian density estimates from other forest types and under different management.
• Forest habitat conditions, including tree species, basal area, and ordinal estimates of vegetative cover, characterize habitat surveyed and which thereby provide benchmarks for bird detections in relation to bottomland hardwood forests of varying structural characteristics.

## 1 Data

**Fig. 1:**
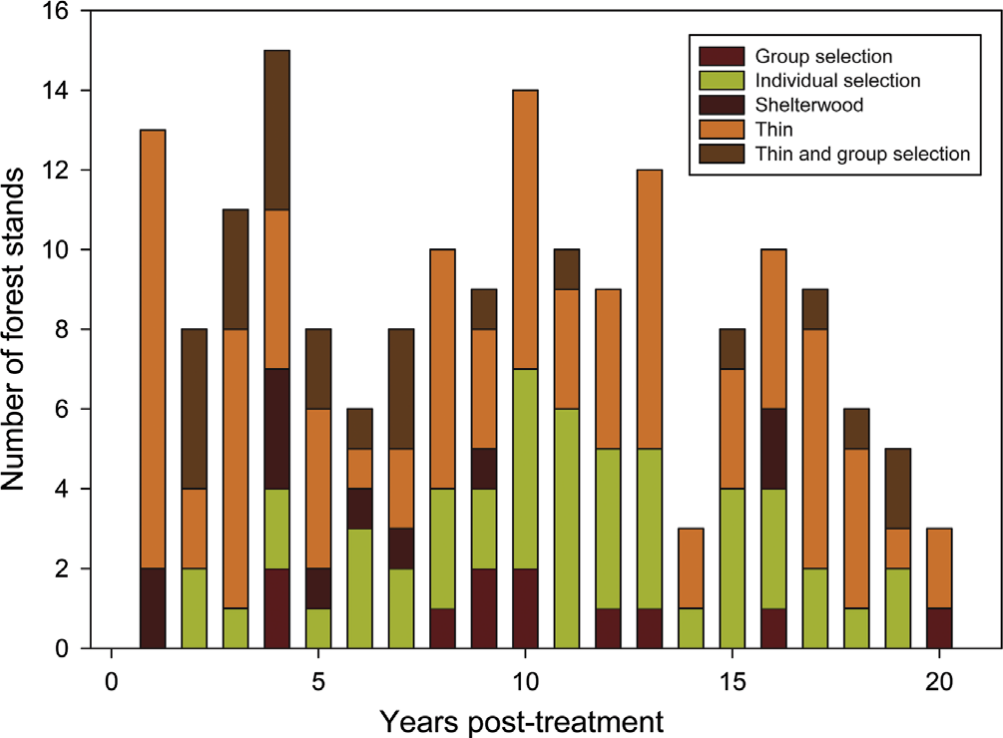
Number of surveyed forest stands in the Mississippi Alluvial Valley surveyed for breeding birds after being subjected to different types of silvicultural treatments and the number of years elapsed between treatment and bird surveys.

The data presented herein were collected during avian counts at point locations on public conservation lands in or proximate to the Mississippi Alluvial Valley, within Arkansas, Louisiana, Mississippi, and Tennessee, USA (Supplementary Table 1). Locations were in forests stands subjected to a range of intensity of silvicultural treatments and number of years post-harvest (Fig. 1). The avian dataset (Supplementary Table 2) provides species, distance (within 4 categorical distance radii), and time (within 3 time intervals) of first detection of each identified bird. The habitat dataset (Supplementary Table 3) provides information on categorical vegetation cover as well as species and diameter class of trees that were within 10 BAF (square feet/acre basal area factor) prism plots that were associated with locations of avian counts. Analyses of these data are presented in the associated research article [5].

## 2 Experimental design, materials and methods

### 2.1 Study areas

Within each of four states (Arkansas, Louisiana, Mississippi, and Tennessee), we surveyed birds on up to five public conservation management areas (National Wildlife Refuge, Wildlife Management Area, or National Forest) during each year of study (2006–2012). On each public conservation area, our experimental units were forest stands on which silvicultural treatment was prescribed for the entirety of the stand, even if treatment was not uniform throughout the stand. Year of treatment was the year treatment was initiated. Experimental stands were treated within the past 20 years (Fig. 1), whereas control stands had not been subjected to silvicultural treatment within the past 20 years - typically not since coming under public management. Local managers subjectively chose control stands with preference for stands of similar forest type to treated stands and included stands designated as natural areas or ‘old-growth areas’.

### 2.2 Bird surveys

**Fig. 2:**
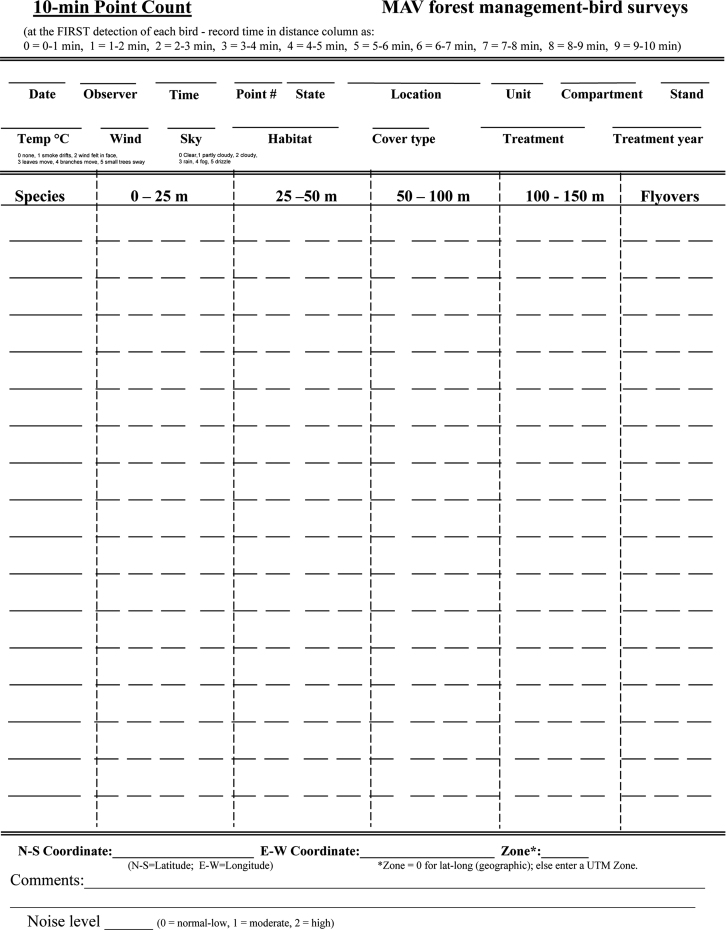
Field data form used to record detections of birds during 10-minute duration counts within forest stands in the Mississippi Alluvial Valley, 2006–2012.

Within each selected forest stand, birds were surveyed at up to six sampling locations that were systematically located 250-meters apart from a random start location and were >100‐m from a primary road or an agricultural edge (Supplementary Table 1). Between 15 May and 30 June, sample locations were surveyed, during clement weather (i.e., no rain or excessive wind) by an experienced observer who recorded bird detection data (Table 1) using a standard field data collection form (Fig. 2). Observers recorded the first detection of each bird within radial distance bands of 0–25 m, >25–50 m, >50–100 m, and >100–150 m, and within time intervals of 0–3 min, >3–5 min, and >5–10 min (Supplementary Table 1).

### 2.3 Habitat surveys

**Fig. 3:**
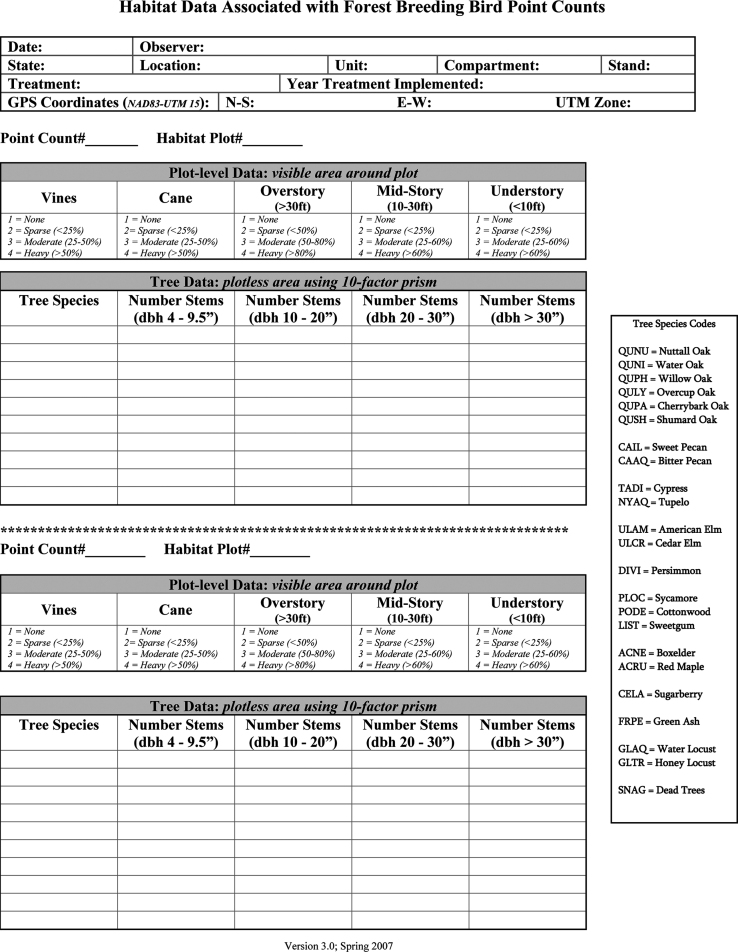
Field data form used to record habitat conditions within 10 BAF (square feet/acre basal area factor) prism plots that were associated with avian point counts in the Mississippi Alluvial Valley, 2006–2012.

Using variable radius plots based on a 10 (square feet/acre) basal area factor (BAF) prism [1], we assessed habitat at two plots associated with each bird survey location: 1 plot at the point and another at approximately 100 m from the point, except in 2006 when only a single plot was sampled. At each habitat plot, we recorded data using a standard field data collection form (Fig. 3) to record habitat (Table 2) including the species and diameter at breast height of each tree within the 10 BAF plot within four size classes: (10–<25 cm, 25–<50 cm, 50–76 and >50 cm, and >76 cm). We also recorded visually discernable cover on an ordinal scale (1=none, 2=sparse, 3=moderate, or 4=heavy) for: vines and cane at 0, >0 <25, 25–50, >50%; understory (<3 m in height) and midstory (3–9 m) at 0, >0 <25, 25–60, >60%; and overstory canopy (>9 m) at 0, >0, <50, 50–80, >80% (Supplementary Table 3).

## Figures and Tables

**Table 1:** Descriptors of data collected during avian point counts in the Mississippi Alluvial Valley, 2006–2012.

Station	Conservation management area. Typically a National Wildlife Refuge (NWR), Wildlife Management Area (WMA), or National Forest.
Unit	Sub-division of management area.
Compartment	Forest management compartment.
Stand	Surveyed stand within compartment - This was the area to which treatments were applied and thus the Experimental Unit of study.
Point	Designation of bird survey count location
Date	Date of survey (year-month-day)
StartTime	Start Time of Bird Survey Point Count (nearest minute on 24 h clock)
Species	Four-letter (English name) Alpha Codes of bird species in accordance with the 57th AOU Supplement (2016) http://www.birdpop.org/pages/birdSpeciesCodes.php
D25_0_3min	Number of detections of the species within 25 m of survey point during first 3 min of survey.
D25_4_5min	Number of detections of the species within 25 m of survey point during minutes 4–5 of survey.
D25_6_10min	Number of detections of the species within 25 m of survey point during minutes 6–10 of survey.
D50_0_3min	Number of detections of the species at distance >25 but <50 m from survey point during first 3 min of survey.
D50_4_5min	Number of detections of the species at distance >25 but <50 m from survey point during minutes 4–5 of survey.
D50_6_10min	Number of detections of the species at distance >25 but <50 m from survey point during minutes 6–10 of survey.
D100_0_3min	Number of detections of the species at distance >50 but <100 m from survey point during first 3 min of survey.
D100_4_5min	Number of detections of the species at distance >50 but <100 m from survey point during minutes 4–5 of survey.
D100_6_10min	Number of detections of the species at distance >50 but <100 m from survey point during minutes 6–10 of survey.
D150_0_3min	Number of detections of the species at distance >100 but <150m from survey point during first 3 min of survey.
D150_4_5min	Number of detections of the species at distance >100 but <150 m from survey point during minutes 4–5 of survey.
D150_6_10min	Number of detections of the species at distance >100 but <150 m from survey point during minutes 6–10 of survey.

**Table 2:** Descriptors of data collected at 10 BAF (square feet/acre basal area factor) prism plots that were associated with avian point counts in the Mississippi Alluvial Valley, 2006–2012.

Station	Conservation management area. Typically a National Wildlife Refuge (NWR), Wildlife Management Area (WMA), or National Forest.	
Unit	Sub-division of management area.	
Compartment	Forest management compartment	
Stand	Surveyed stand within compartment - This was the area to which treatments were applied and thus the Experimental Unit of study.	
Point	Designation of Bird Survey Point Count location with which vegetation plot(s) are associated	
Date	Date of survey (year–month–day)	
StartTime	Start Time of Bird Survey Point Count (nearest minute on 24 h clock)	
HabitatPlot	Designation of Vegetation Plot associated with Bird Survey Point Count (1 or 2 plots were associated with each Bird Survey Point Count).	
Observer	Observer	
Treatment	Descriptive designation of treatment as applied by operational forester.	
TrmtYear	Year treatment commenced.	
Vine	Ordinal scale (1=none, 2=sparse, 3=moderate, or 4=heavy): category percentages of ordination scale were 0%, >0–<25%, 25–50%, >50%.	
Cane	Ordinal scale (1=none, 2=sparse, 3=moderate, or 4=heavy): category percentages of ordination scale were 0%, >0–<25%, 25–50%, >50%.	
Understory (<3 m in height)	Ordinal scale (1=none, 2=sparse, 3=moderate, or 4=heavy): category percentages of ordination scale were 0%, >0–<25%, 25–60%, >60%.	
Mid_story (3–9 m in height)	Ordinal scale (1=none, 2=sparse, 3=moderate, or 4=heavy): category percentages of ordination scale were 0%, >0–<25%, 25–60%, >60%.	
Overstory (>9 m in height)	Ordinal scale (1=none, 2=sparse, 3=moderate, or 4=heavy): category percentages of ordination scale were 0%, >0–<50%, 50–80%, >80%.	
TreeSps	4 letter alpha code designation for tree species Genus (2 letters) and species (2 letters) - Species are listed below:	
BasalArea	Basal Area of the species within the 10 BAF prism plot (i.e., 10x number of stems ׳in׳ plot)	
dbh_LT10	Number of trees of the species ׳in׳ the 10 BAF prism plot with diameters at breast height (dbh) that were >4 inches and <10 inches; (>10 cm–24 cm)	
dbh_10_20	Number of trees of the species ׳in׳ the 10 BAF prism plot with diameters at breast height (dbh) that were >10 inches and <20 inches; (25–50 cm)	
dbh_20_30	Number of trees of the species ׳in׳ the 10 BAF prism plot with diameters at breast height (dbh) that were >20 inches but <30 inches; (51–76 cm)	
dbh_GT30	Number of trees of the species ׳in׳ the 10 BAF prism plot with diameters at breast height (dbh) that were >30 inches; (>76 cm)	
Alpha Code	Species name	Common name
–	(no trees in 10 BAF plot)	–
ACNE	*Acer negundo*	Boxelder
ACRU	*Acer rubrum*	Red maple
ACSA	*Acer saccharinum* or *Acer saccharum*	Silver maple or sugar maple
ASTR	*Asimina triloba*	Pawpaw
BENI	*Betula nigra*	River birch
CAAQ	*Carya aquatica*	Bitter pecan
CACA	*Carpinus caroliniana*	Musclewood, hornbeam
CAIL	*Carya illinoensis*	Sweet pecan
CAOV	*Carya ovata*	Shagbark hickory
CARY	*Carya* species	Unidentified hickory
CATO	*Carya tomentosa*	Mockernut hickory
CELA	*Celtis laevigata*	Sugarberry
CODR	*Cornus drummondii*	Rough-leafed dogwood
COFL	*Cornus florida*	Flowering dogwood
COSP	*Cornus* species	Unidentified dogwood
CRSP	*Crataegus* species	Unidentified hawthorn
DIVI	*Diospyros virginiana*	Persimmon
FOAC	*Foresteria acuminata*	Swamp foresteria, swamp privet
FRPE	*Fraxinus pennsylvanica*	Green ash
GLAQ	*Gleditsia aquatica*	Water locust
GLED	*Gleditsia* species	Unidentified locust
GLTR	*Gleditsia triacanthos*	Honey locust
ILDE	*Ilex decidua*	Possumhaw, deciduous holly
JUVI	*Juniperus virginiana*	Eastern red cedar
LIST	*Liquidambar strasyflua*	Sweet gum
LITU	*Liriodendron tulipifera*	Tulip tree
MORU	*Morus rubra*	Red mulberry
NYAQ	*Nyssa aquatica*	Water tupelo
NYSY	*Nyssa sylvatica*	Black gum
OSVI	*Ostrya virginiana*	Ironwood, hop hornbeam
PITA	*Pinus taeda*	Loblolly pine
PLAQ	*Planera aquatica*	Water elm, planertree
PLOC	*Platanus occidentalis*	American sycamore
PODE	*Populus deltoides*	Cottonwood
PRSE	*Prunus serotina*	Black cherry
QUAL	*Quercus alba*	White oak
QUER	*Quercus species*	Unidentified oak
QUFA	*Quercus falcata*	Southern red oak
QULA	*Quercus laurifolia*	Laurel oak
QULY	*Quercus lyrata*	Overcup oak
QUMI	*Quercus michauxii*	Cow oak, swamp chestnut oak
QUNI	*Quercus nigra*	Water oak
QUNU	*Quercus nuttalli, Quercus texana*	Nuttall׳s oak
QUPA	*Quercus pagoda*	Cherrybark oak
QUPH	*Quercus phellos*	Willow oak
QUSH	*Quercus shumardii*	Shumard oak
QUST	*Quercus*	Unidentified oak
QUVE	*Quercus velutina*	Black oak
RED	(*Lobatae*) red oak group species	Unidentified ׳red׳ oak
ROPS	*Robinia pseudoacacia*	Black locust
SAAL	*Sassafras albidum*	Sassafras
SANI	*Salix nigra*	Black willow
SASE	*Sapium sebifera, Triadica sebifera*	Tallow tree
SNAG	(dead tree)	Unidentified dead tree
TADI	*Taxodium distichum*	Blad cypress
ULAL	*Ulmus alata*	Winged elm
ULAM	*Ulmus americana*	American elm
ULCR	*Ulmus crassifolia*	Cedar elm
ULMU	*Ulmus* species	Unidentified elm
ULRU	*Ulmus rubra*	Slippery elm, red elm
UNKN	unkown	Unidentified species
WHIT	(*Quercus*) white oak group species	Unidentified ׳white׳ oak

## Footnotes

Appendix A Supplementary data associated with this article can be found in the online version at http://dx.doi.org/10.1016/j.dib.2016.11.01.
